# The Death of the Emperor of China

**Published:** 1909-06-12

**Authors:** 


### THE DEATH OF THE EMPEROR OF CHINA.

The Edicts and Memorials issued in connection with the death of their Imperial Majesties the Emperor of China and the Great Empress Dowager contain some curious and almost amusing reading.

An Imperial Edict issued on November 14, 1908, runs as follows:—

From the beginning of autumn of last year our health has been poor. We ordered the Tartar Generals, Viceroys and Governors of every province to recommend physicians of ability. Thereupon the Viceroys of Chilhi, the Liang Kiang, Kiangsu and Chekiung recommended and sent forward Ch'en Ping Chun, Ts'ac Yuan Weng, Lu Yung Pin, Chou Ching Tae, Tu Chung Chun, Shih Huan, and Chang P'ang Nien, who came to Pekin and treated us but their prescriptions have given us no relief. Now the negative and positive elements (Yin Yang) are both failing. There are ailments both external and internal, the breath is stopped up, the stomach is rebellious, the back and legs painful, appetite failing. On moving, the breath fails, and there is coughing and panting. Besides, we have chills and fever, cannot sleep at night, experience a general failure of bodily strength which is hard to bear. Our heart is very impatient and now the Tartar Generals, Viceroys and Governors of every province are ordered to select capable physicians regardless of their official rank and send them quickly to Pekin to await summons to give medical aid. If any can show beneficial results he will receive extraordinary rewards and the Tartar Generals, Viceroys or Governors who recommend him will receive special grace. Let this be published.

In spite of the efforts extended on behalf of the suffering Empress by the physicians named, her Majesty "ascended on the dragon to be a guest on high." Four days later a further edict was issued in which these practitioners were "degraded two steps and left in office"! Certain other physicians were "degraded and will work without stipends as punishment." Note the above are the regular palace physicians.

